# Environmental pressures shape regional patterns of genetic diversity and ancestry in cotton landraces

**DOI:** 10.3389/fpls.2025.1707011

**Published:** 2025-11-21

**Authors:** Avinash Shrestha, Maximus A. Gudino, Rosalyn B. Angeles-Shim

**Affiliations:** Department of Plant and Soil Science, Davis College of Agricultural Sciences and Natural Resources, Texas Tech University, Lubbock, TX, United States

**Keywords:** *Gossypium hirsutum*, selective sweep, population structure, photoperiod sensitivity, genetic variation

## Abstract

Upland cotton has undergone extensive domestication and breeding, leading to substantial genetic improvement but also a pronounced narrowing of its genetic base. To better characterize and leverage the diversity preserved in traditional gene pools, we examined the population structure, phylogenetic relationships, and genomic signatures of selection in a globally sourced panel of cotton landraces and elite cultivars. STRUCTURE and neighbor-joining analyses based on whole-genome SNP genotyping identified four ancestral populations divided into nine major clusters. The landrace accessions formed deep, regionally coherent lineages characterized by high heterozygosity and an abundance of private alleles. Consistent with these patterns, Nei’s genetic distance and pairwise F_ST_ estimates revealed strong divergence between Mesoamerican and Central American landraces relative to modern breeding lines. Flowering time, a key adaptive trait, was strongly associated with genetic clusters, with photoperiod-sensitive genotypes primarily originating from highland and tropical regions. Genome-wide scans of Tajima’s D further differentiated landraces from cultivars, revealing signatures of balancing selection and ancestral polymorphism in the landraces, and selective sweeps in cultivated accessions. Notably, flowering-related genes on chromosomes D05 and A05 were located in regions exhibiting contrasting Tajima’s D values between the two gene pools. These findings demonstrate that cotton landraces have retained valuable genomic regions lost from modern cultivars through domestication and decades of intensive improvement. As such, they represent an important reservoir for enhancing resilience, adaptation, and fiber traits in modern cotton. Collectively, our results provide a high-resolution framework for targeted pre-breeding and conservation initiatives, underscoring the untapped potential of landraces in broadening the genetic base of cultivated *G. hirsutum*.

## Introduction

Cotton (*Gossypium hirsutum* L.) is among the most extensively cultivated fiber crops worldwide, with a long history of domestication and cultivation across diverse ecological regions. Its production supports major agricultural economies, particularly in the United States, China, India, and Pakistan. Over centuries, selective breeding in cotton has focused primarily on enhancing fiber yield and quality, along with improving resistance to pests. While this resulted in the production of elite cultivars tailored for industrial-scale production, the narrow focus on agronomic performance has inadvertently led to a reduction in the crop’s genetic diversity.

Like most crop improvement programs, cotton breeding has relied heavily on “good-by-good” crossing strategies, repeatedly interbreeding elite lines with similar genetic backgrounds. As a result, contemporary cultivars exhibit overlapping pedigrees and a progressively constricted genetic base. Analyses of over 100 upland cotton genotypes developed between the 1800s and 1990s revealed minimal genetic differentiation among regional breeding pools (Lubbers et al., 2005). In 2020, U.S. cotton production relied on just 29 commercial varieties, many of which shared common parentage (USDA, 2022; Lubbers and Chee, 2009). These patterns reflect a long-standing and persistent trend toward genetic uniformity in cultivated cotton.

This erosion of genetic diversity has tangible consequences for cotton’s long-term agronomic sustainability. Genetically uniform crop cultivars tend to be more vulnerable to biotic and abiotic stressors, with their limited genetic foundation constraining the crop’s adaptive potential under changing climatic conditions. Recent events have underscored these risks for cotton. For example, in 2017, an outbreak of *Fusarium oxysporum* f. sp. *vasinfectum* race 4 (FOV4) severely impacted cotton production in Texas, where resistant cultivars were unavailable (Ulloa et al., 2020). Similarly, the 2011 drought led to a sharp decline in cotton acreage across the southern United States, exposing the crop’s vulnerability to water stress. In other major cotton-producing regions such as China and Pakistan, recurring floods, heat waves, and pest outbreaks have further demonstrated the limitations of genetically narrow cultivars in coping with environmental extremes (Guerrero, 2013; Qian et al., 2020; Ullah et al., 2017). Without access to a broader pool of genetic variation, breeding programs face restricted opportunities to introduce novel alleles that confer resilience, placing the future viability of cotton cultivation at risk.

Landraces or traditional crop varieties that have been maintained outside of formal breeding systems offer a promising avenue for restoring diversity in cotton. Genetically heterogeneous and shaped by long-term cultivation under diverse environmental conditions, these populations have evolved through farmer selection rather than controlled breeding. As a result, they often exhibit region-specific ancestry and retain alleles associated with adaptive fitness such as tolerance to soil salinity, water stress, and plant pests and diseases (Mercer and Perales, 2010; Lazaridi et al., 2024). Like the wild relatives of crop species, landraces harbor naturally occurring genetic variation that has been filtered through generations of environmental and cultural selection. This preconditioned variation, shaped by real-world selective pressures acting on organismal fitness, is widely regarded as a more reliable source of adaptive traits than artificially induced mutations (Mangat et al., 2021). Accordingly, landraces represent especially valuable reservoirs of resilience-enhancing alleles for modern cotton breeding.

The potential of landraces to enhance genetic diversity and resilience has been well documented across multiple crop species. In rice, for example, landraces have contributed key adaptive alleles such as *Sub1A* for submergence tolerance, *Saltol* for salinity tolerance, and *pi21* for blast resistance (Angeles-Shim et al., 2020; De Leon et al., 2017; Krishnamurthy et al., 2020). In wheat, landraces have provided resistance to a broad range of biotic and abiotic stresses, including stem rust and drought (Lopes et al., 2015). In cotton, various studies identified tolerance to drought and cold stress of landraces (Hou et al., 2018; Shim et al., 2019), although their broader genomic potential remains largely unexplored. Collectively, these examples underscore the value of landraces as sources of genetic innovation and adaptive traits for crop improvement.

Despite their promise, cotton landraces remain underutilized in breeding programs. Barriers such as photoperiod sensitivity, agronomically disadvantageous traits, and limited genomic and phenotypic characterization have hindered their integration into modern cultivars (Campbell et al., 2019; Shim et al., 2021). Although recent efforts to develop day-neutral conversion lines are beginning to address these limitations (McCarty et al., 1996, 2006), much of the genetic potential within landraces remains untapped.

To address this gap, we assessed the natural genetic variation and investigated how specific environmental pressures have shaped regional patterns of diversity and ancestry in a panel of cotton landraces. Specifically, we aimed to (1) characterize population structure and ancestry relationships across landraces from ecologically distinct regions, (2) identify genomic regions in the landraces that are associated with local adaptation, and (3) evaluate the role of environmental variables in shaping genetic differentiation. By examining these patterns, our study provides a foundation for future efforts to link naturally occurring variation with agronomic traits such as drought and cold tolerance. Ultimately, this work supports the strategic use of regionally adapted germplasm to enhance cotton’s resilience and long-term performance under variable environmental conditions.

## Materials and methods

### Plant materials

**Figure 1:**
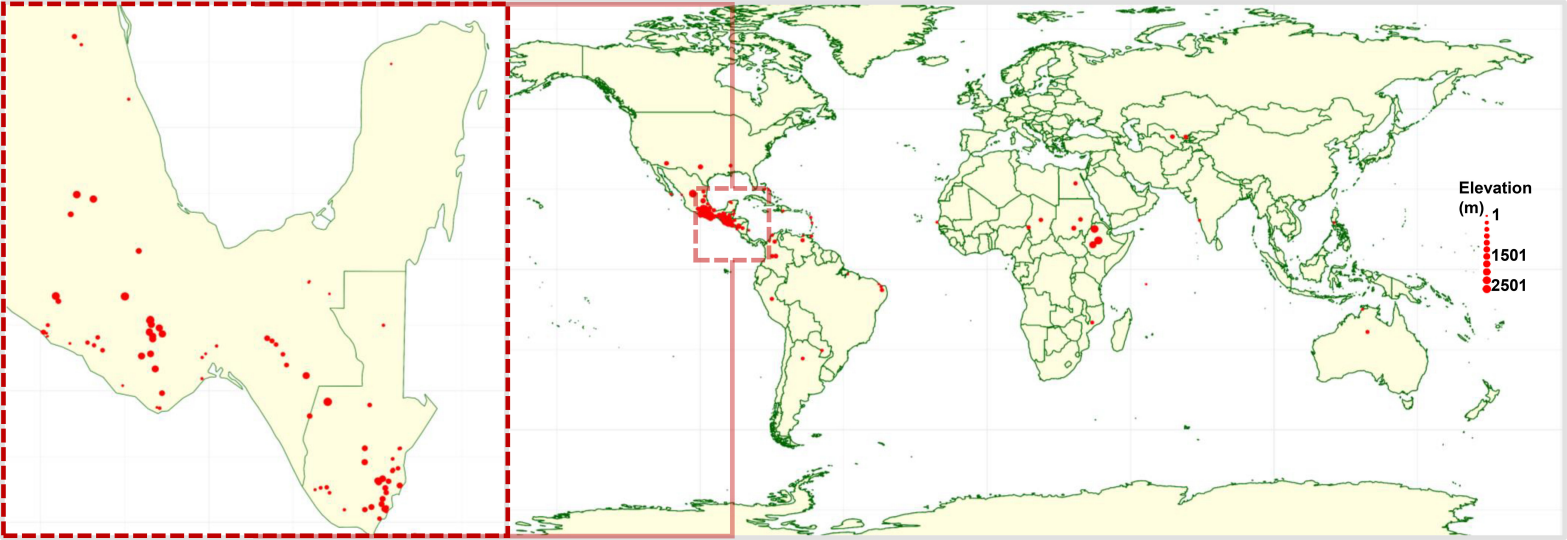
Global geographic distribution of accession in the landrace panel. Each red circle represents a landrace collection site, with circle size corresponding to the elevation of the site, ranging from sea level up to 2,500 meters above sea level.

A panel of 380 *Gossypium hirsutum* accessions composed of 374 landraces and six elite cultivars (DP2020, ST4553, FA1370, FM1380, FM2398, and TM-1) was assembled and used for this study. The elite lines represent widely adopted commercial cultivars with combined herbicide tolerance, insect resistance, high yield potential, and superior fiber quality. The landrace accessions were selected to capture broad geographic representation across the Americas, Africa, Asia, and the Caribbean (**Figure 1**), as well as genetic variation in key agronomic traits, including seed oil content and photoperiod sensitivity.

Seeds were sourced from the USDA Germplasm Resources Information Network (USDA-GRIN) (https://www.ars-grin.gov/). For each accession, passport data including country of origin, latitude, longitude, elevation, a USDA PI numbers were compiled. Trait information, including photoperiod sensitivity, was compiled from the USDA-GRIN database, which integrates records collected across different sites and years; therefore, these data were used cautiously and interpreted only for broad comparative purposes. Geographical coordinates were verified, and missing elevation data were retrieved using the Google Maps Geocoding Application Programming Interface.

The landrace accessions were germinated in BM6 soil mix and grown in trays under controlled greenhouse conditions (30°C day/28°C night) at Texas Tech University.

### DNA extraction and SNP genotyping

Leaf tissues were sampled from seedlings at the 2- to 3-leaf stage. Whole genomic DNA was extracted using a modified CTAB protocol, with 2% β-mercaptoethanol added to the buffer to enhance cell lysis and reduce interference from secondary metabolites (Murray and Thompson, 1980). DNA quality was evaluated on 1% agarose gels, and DNA concentrations were quantified using a NanoDrop 2000 spectrophotometer (Thermo Fisher Scientific, Waltham, MA, USA). Only samples with concentrations of ≥1 µg/20 µL and A260/280 ratio between 1.8 and 2.0 were retained for downstream analysis.

Genotyping was conducted at the Texas A&M Institute for Genomic Sciences and Society using the CottonSNP27K array, a curated subset of the CottonSNP63K array containing 27,825 non-redundant SNPs selected for broad genome-wide coverage (Hulse-Kemp et al., 2015). Genotype calling was carried out in GenomeStudio v2.0 (Illumina Inc., https://support.illumina.com) using a cluster file optimized by SGS-TraitGenetics for consistency with the CottonSNP63K platform. SNP calls were exported in FinalReport format, and quality metrics were assessed using default GenomeStudio thresholds and reference line comparisons.

### SNP curation and filtering

Genotype data from the CottonSNP27K array were curated to ensure marker quality for downstream analysis. All data were processed using R v4.2.0 and Excel 2016. SNPs with no genotype calls, more than 20% missing data, or that were monomorphic were removed. Accessions with over 50% missing calls were also excluded to maintain data integrity (Uffelmann et al., 2021; Shrestha et al., 2024; Shrestha, 2025).

The remaining polymorphic SNPs were annotated using the TM-1_CRIV1 reference genome assembly for *Gossypium hirsutum* (Zhang et al., 2015). SNPs with inconsistent or unreliable alignments to the reference genome were excluded to ensure genomic consistency and enable robust comparisons with previous studies.

### Genetic diversity and population structure analysis

#### Model-based population structure analysis

Population structure was inferred using STRUCTURE v2.3.4 (Pritchard et al., 2010). The analysis was conducted using the admixture model with correlated allele frequencies, which is appropriate for populations with shared ancestry. Analyses were conducted for K values ranging from 1 to 10, with five independent runs per K. Each run included a burn-in period of 5,000 iterations followed by 5,000 MCMC repetitions commonly used for large SNP datasets to ensure convergence and reliable ancestry estimation (Porras-Hurtado et al., 2013).

STRUCTURE outputs were processed using CLUMPAK (http://clumpak.tau.ac.il), which generated consensus Q-matrices and population bar plots, and identified the optimal K based on the ΔK method (Evanno et al., 2005; Kopelman et al., 2015). The corresponding Q-matrix was extracted and used for downstream classification of individuals. Individuals with a membership probability ≥60% for a single cluster were assigned to that population. Accessions with no single cluster exceeding the threshold were classified as admixed but retained for visualization and interpretation of introgression patterns (Porras-Hurtado et al., 2013).

The final Q-matrix was imported into R v4.2.0 for downstream analyses. Genotype data were converted to *genind* and *genpop* objects using the adegenet package (Jombart, 2008). Nei’s genetic distances between STRUCTURE-defined groups were calculated using the *poppr::nei.dist()* function, and within-group genetic variability was assessed via average pairwise Euclidean distances computed from mean-imputed SNP matrices using the *ade4::dist()* function (Kamvar et al., 2014).

#### Neighbor-joining tree construction

Genetic variation within the landrace panel was assessed using a series of complementary analyses conducted using TASSEL and R-based workflows (Bradbury et al., 2007). The analysis aimed to provide insights into genetic relationships, clustering patterns, and potential subpopulation differentiation within the curated panel.

Phylogenetic relationships among the 380 *G. hirsutum* accessions were investigated using a neighbor-joining (NJ) tree constructed from pairwise identity-by-state (IBS) genetic distance matrices. The analysis was carried out with a filtered SNP dataset (18,258 SNPs), which was converted into HapMap format and loaded into TASSEL v5.2.77 (Bradbury et al., 2007). IBS distances were computed using the ‘Genetic Distance Matrix’ function in TASSEL, specifying the identity-by-state method with default options. Prior to tree construction, missing data were imputed using the mean genotype value per marker. The resulting distance matrix was saved as a tab-delimited file and processed through TASSEL’s NJ tree generator to produce a Newick-formatted tree.

The resulting Newick-formatted (.nwk) tree file was exported and uploaded to the Interactive Tree of Life (iTOL) platform for graphical visualization and annotation (Letunic and Bork, 2021). Branches were color-coded by geographic origin and accession type (landrace vs. cultivar) to assess congruence between genetic clustering and passport data.

#### Tajima’s D and genome-wide evolutionary analysis

To assess genome-wide patterns of nucleotide variation and identify regions potentially under selection, Tajima’s D was calculated in TASSEL using a sliding window approach (Tajima, 1989). The analysis was conducted separately for two genotype subsets: one composed of landrace accessions, and another consisting exclusively of cultivated lines. This comparative framework was designed to reveal distinct evolutionary pressures acting on traditionally maintained populations versus modern breeding materials. Tajima’s D values were computed across the genome using a window size of 50 SNPs and a step size of 200 base pairs, enabling fine-scale resolution of selective sweeps and localized signals while maintaining statistical reliability (Carlson et al., 2005). Output files were imported into R for processing and visualization using the tidyverse suite, including dplyr, readr, and ggplot2 (Wickham, 2025). Chromosomes were ordered and annotated based on the TM-1 reference genome (Zhang et al., 2015), and midpoint positions were calculated for plotting. Tajima’s D values were visualized chromosome by chromosome using *facet_wrap()* in ggplot2, and comparative line plots were generated to highlight differences in selection signatures between landraces and cultivars. Positive Tajima’s D values suggest balancing selection or population contraction, while negative values indicate directional or purifying selection or historical population expansion. All results were interpreted in the context of population structure, geographic origin, and potential domestication-related signatures.

## Results

### Geographic distribution and key traits of landrace accessions

Most of the landrace accessions in the panel originated from regions within Mesoamerica which are historically recognized as centers of diversity and primary sites of upland cotton domestication (Smith and Cothren, 1999; Vavilov and Dorofeev, 1992) (**Supplementary Table S1**). Notably, over 80% of the accessions were sourced from Mexico (182 accessions) and Guatemala (130 accessions). The remaining accessions were obtained from Belize, Colombia, and the United States, contributing to the panel’s broad geographic representation (**Figure 1**).

The native habitats of these accessions span a wide elevation range from as low as 5 meters to as high as 3,510 meters above sea level, with a mean elevation of 1,529 meters. This extensive altitudinal distribution reflects the adaptive capacity of landraces to diverse agro-ecological zones and likely underpins the phenotypic and genetic variation observed across the panel (**Supplementary Table S1**).

Available photoperiod response data for almost all accessions in the panel revealed substantial variation in flowering behavior. A total of 179 accessions were classified as photoperiod-insensitive and capable of flowering within a single growing season. Conversely, 190 were photoperiod-sensitive, requiring extended seasonal exposure for floral induction.

### Stringent SNP curation reveals a high-quality genome-wide marker set for diversity analysis

Quality assessment of the returned genotype data removed 2,027 SNPs with universally missing genotype calls and 58 SNPs with more than 80% missing data. Additionally, 170 monomorphic loci were excluded to retain only informative, segregating markers.

Among the remaining markers, 2,221 could not be mapped to any chromosome on the TM-1_CRIV1 reference genome and were therefore excluded from the analysis. The final dataset consisted of 18,258 high-confidence SNPs representing 66.9% of the original marker set. These SNP were distributed across all 26 chromosomes of the A and D sub-genomes in 379 landraces accessions. None of the markers amplified in accession 226L, which was consequently excluded from downstream analysis.

Chromosome-wise distribution of SNPs demonstrated robust genome coverage. In the A sub-genome, SNP counts ranged from 364 on A04 to 1,083 on A08 followed by A05 (850 SNPs) and A13 (868 SNPs). In the D sub-genome, D05 (1,267 SNPs) and D08 (1,046 SNPs) exhibited the highest marker densities, while D03 (467), D04 (429), and D11 (575) had the lowest. SNP positions spanned the full length of each chromosome, with start coordinates ranging from 480 bp on D06 to over 125 Mbp on A08 (**Supplementary Figures S1a-b**).

The average call rate across all retained SNPs was greater than 97%. The mean polymorphic information content (PIC) for the markers was 0.32. The minor allele frequency (MAF) distribution showed that 13.2% of the SNPs had MAF < 0.05; 68.5% had MAF values between 0.10 to 0.45; and approximately 2.4% had MAF values greater than 0.45 (**Supplementary Figure S2**).

### Principal component and K-means clustering partition landraces by geographic origin

**Figure 2:**
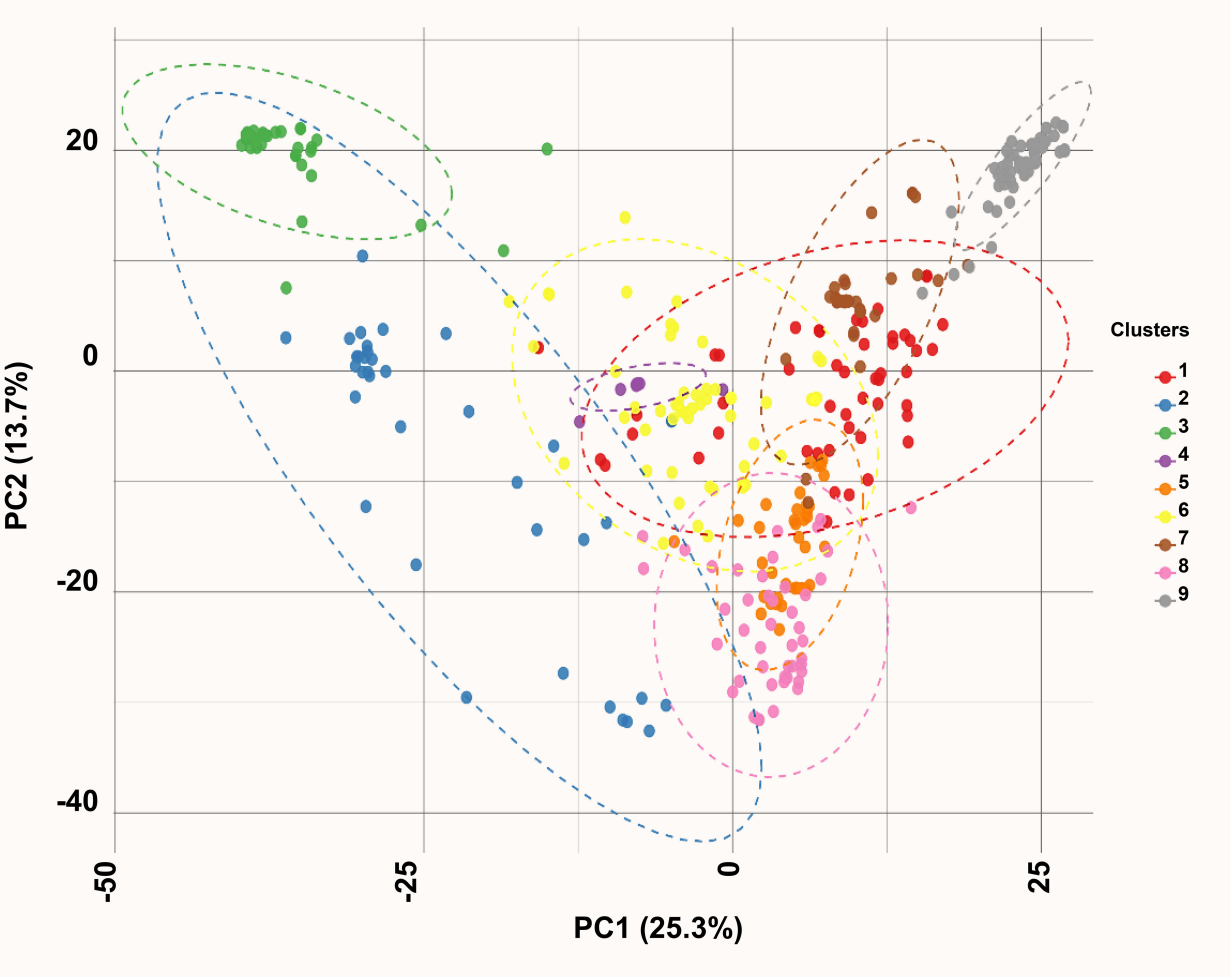
Principal component analysis depicting nine genetically distinct clusters.

Principal component (PC) analysis of the 18,258 curated SNPs revealed distinct genetic structure within the diversity panel. The first two principal components accounted for 39% of the total genetic variance. Accessions were projected onto the PC1–PC2 plane, revealing three well-defined clusters corresponding to Clusters 2, 3, and 9. Cluster 2 comprised accessions from Central America and southern Mexico, Cluster 3 included landraces from northern and central regions, and Cluster 9 was dominated by accessions from Chiapas, Mexico, and the Guatemalan highlands. A set of admixed accessions occupied intermediate positions between these clusters (**Figure 2**, **Supplementary Table S1**).

Unsupervised K-means clustering using the first five PCs optimally partitioned the panel into nine genetic groups (K-means Groups I–IX), as determined by the elbow criterion. Group IX consisted predominantly of Central American accessions, especially those from Jalapa and Chiquimula, Guatemala. Groups I, II, and VI were dominated by North-American landraces, whereas Groups III, IV, V, VII, and VIII included accessions from North, Central, and South America, with several Asian accessions intermixed.

### STRUCTURE analyses reveal four distinct subpopulations in the landrace panel

**Figure 3:**
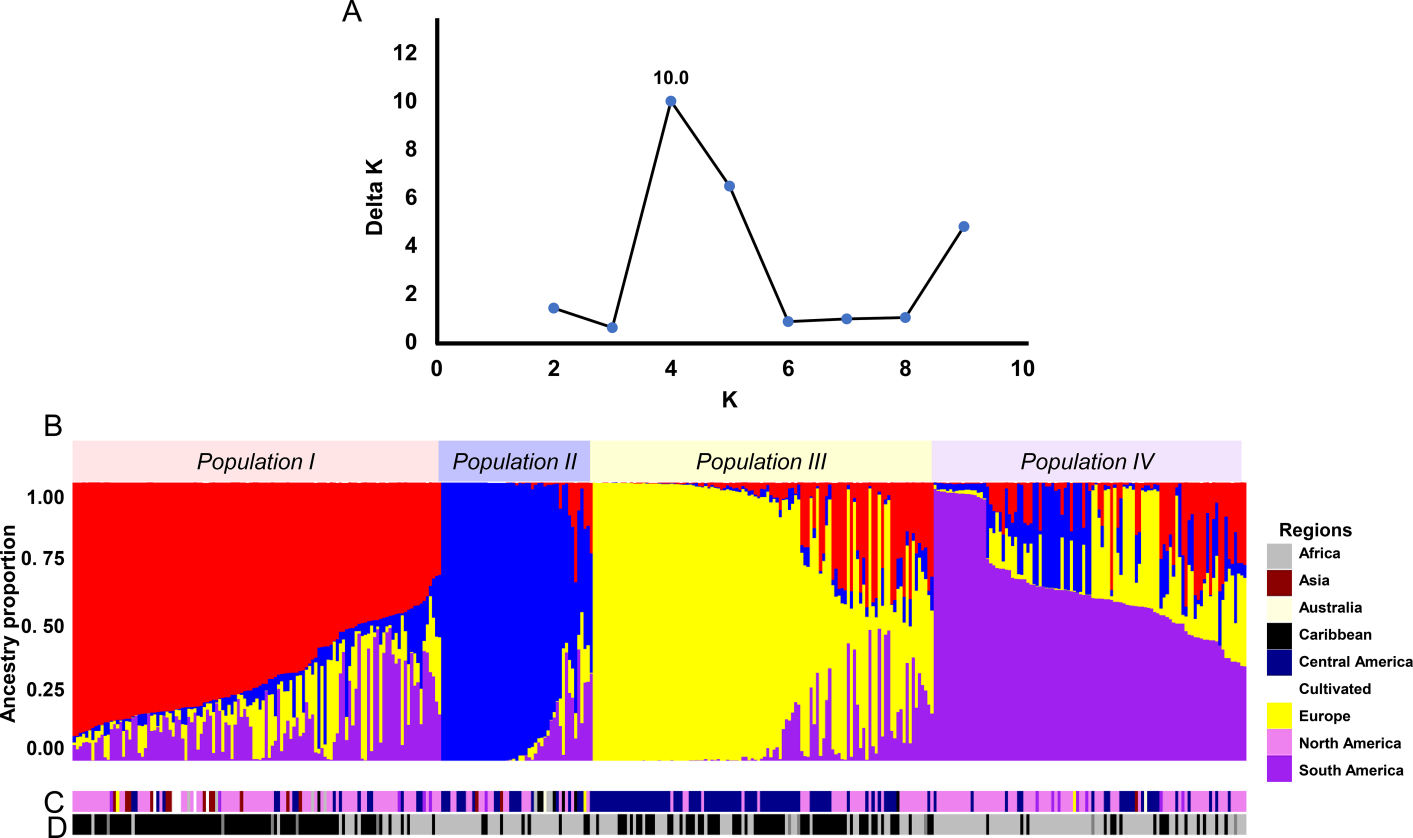
Results of the STRUCTURE analysis. **(A)** Determination of the optimum number of genetic clusters (K) using the ΔK method, as identified by CLUMPAK. **(B)** Ancestry proportion plot of individual landraces at K=4, sorted by Q values. **(C)** Geographic distribution of the individuals in the STRUCTURE population. The inset legend identifies color codes for each population. **(D)** Photoperiod-sensitivity classification of accessions corresponding to STRUCTURE populations, where black represents photoperiod-insensitive and gray represents photoperiod-sensitive lines.

The ΔK method identified a sharp peak at K=4, dividing the landrace accessions into four populations composed of individuals with shared ancestry (**Figure 3A**). This result was supported by four partitions in the STRUCTURE bar plot, where individual genotypes displayed coherent ancestry blocks with varying levels of admixture (**Figure 3B**).

Nei’s genetic distance analysis showed the greatest divergence between Populations I and II (0.2231), followed by Populations II and III (0.2018) (**Table 1**). Pairwise F_ST_ estimates were highest between Populations I and II (F_ST_ = 0.684) and between Populations II and III (F_ST_ = 0.686). In contrast, Populations I and IV exhibited relatively low differentiation (F_ST_ = 0.0909) (**Table 1**).

**Table 1:** Pairwise Nei’s genetic distances and F_ST_ values among STRUCTURE-defined populations of upland cotton.

Populations	Nei’s distance				F_ST_			
	*Pop I*	*Pop II*	*Pop III*	*Pop IV*	*Pop I*	*Pop II*	*Pop III*	*Pop IV*
*Pop I*	0.000	0.223	0.181	0.091	0.000	0.684	0.422	0.091
*Pop II*	0.223	0.000	0.202	0.198	0.684	0.000	0.686	0.532
*Pop III*	0.181	0.202	0.000	0.125	0.422	0.686	0.000	0.328
*Pop IV*	0.091	0.198	0.125	0.000	0.091	0.532	0.328	0.000

Within-group genetic distances also varied across populations. Population I exhibited a mean pairwise genetic distance of 207.96. Population II had the lowest within-group distance at 141.88. Population III displayed a mean within-group distance of 170.76, while Population IV showed the highest internal variation, with a mean genetic distance of 198.98.

### Regional and global distribution patterns revealed through phylogenetic analysis of cotton genotypes

**Figure 4:**
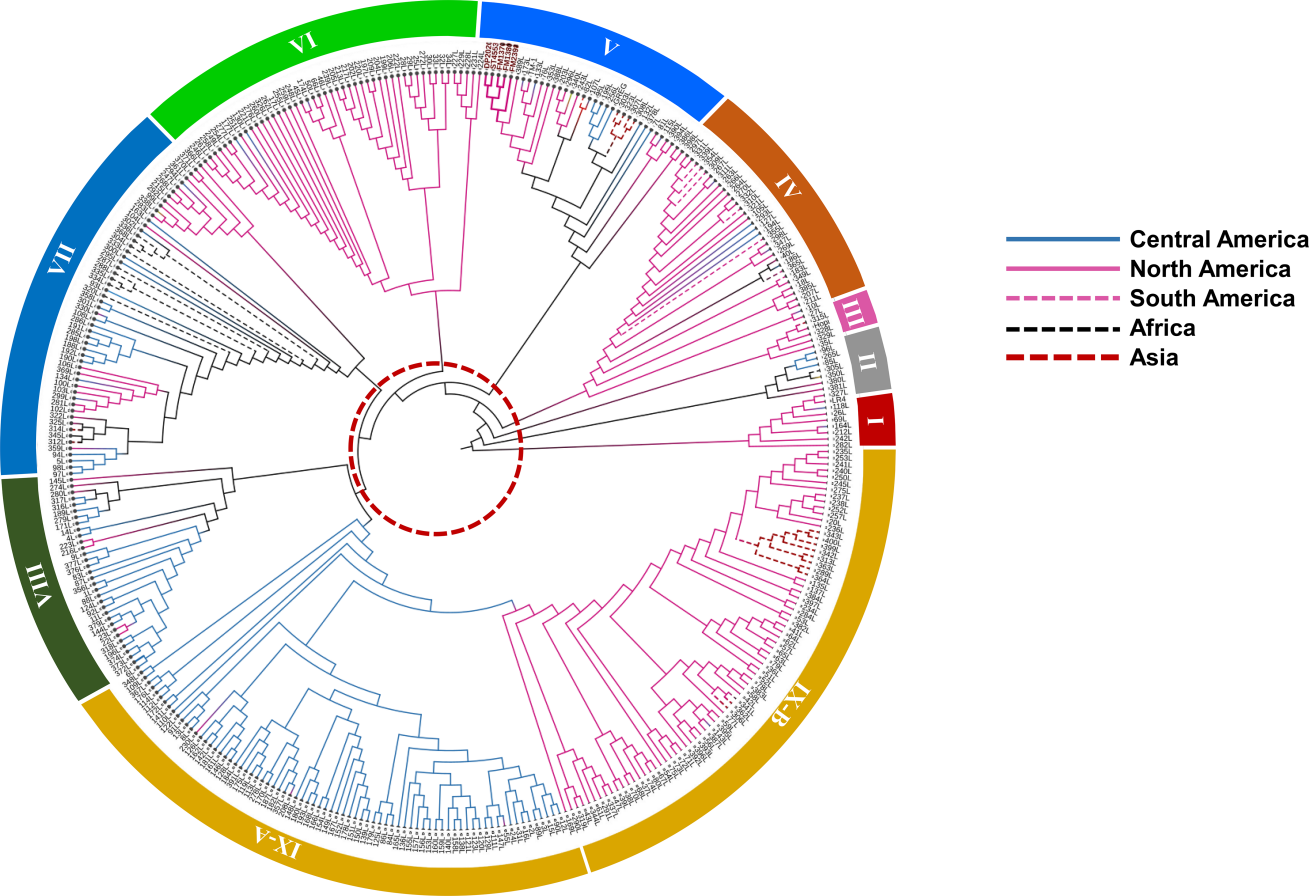
Neighbor-Joining phylogenetic tree depicting genetic relationships among cotton landraces, grouped into nine clusters. Branches are color-coded by geographic region of origin. The red dotted circle highlights a genetic distance threshold of 1.05, used to define major clusters within the tree.

The NJ phylogeny revealed nine major genetic clusters, delineated by internal branch length ranging from 1.09 to 2.84 units (**Figure 4**). The branch thresholds exceeded the average within-group branch length of 0.60 units, indicating well-supported genetic groupings. The clusters were color-coded by continental origin namely blue for Central American accessions, pink for North American accessions, bold pink for the elite cultivars, magenta for South America accessions, black dashed line for African accessions, red dashed line for Asian accessions and yellow for accessions from Oceania (**Figure 4**).

Cluster I consisted of ten Guatemalan and Southern Mexican genotypes, with an average internal divergence of 0.72. Cluster II included seven North American, Central American, and West African genotypes with the highest internal divergence at 0.86 compared to all clusters. Cluster III comprised five genotypes from northern and central regions of the Americas, with a divergence of 0.76. Cluster IV grouped 33 accessions from North, Central, and South America, as well as one from Seychelles, and showed a divergence of 0.71. Cluster V contained 35 genotypes, including all cultivated varieties along with TM-1 and several US landraces. Genotypes in this cluster had the lowest internal divergence at 0.61. Cluster VI was composed of 49 landraces originating from the highlands of Oaxaca, Guerrero, and Chiapas in Mexico, as well as parts of Guatemala and El Salvador, with an average divergence of 0.68. Cluster VII was geographically diverse, encompassing 51 genotypes from Central America, North America, Africa, Asia, and the Caribbean, and showed internal divergence of 0.76. Cluster VIII included 34 accessions largely from the Guatemalan highlands, along with one from Australia, with an internal divergence of 0.83. Cluster IX was the largest, containing 153 accessions from North America, Central America, Asia, and the Caribbean. This cluster was further subdivided into Cluster IX-A and B. IX-A is composed of Central American genotypes with a divergence value of 0.58, whereas IX-B consisted mostly of North American and Asian genotypes with divergence of 0.67.

**Figure 5:**
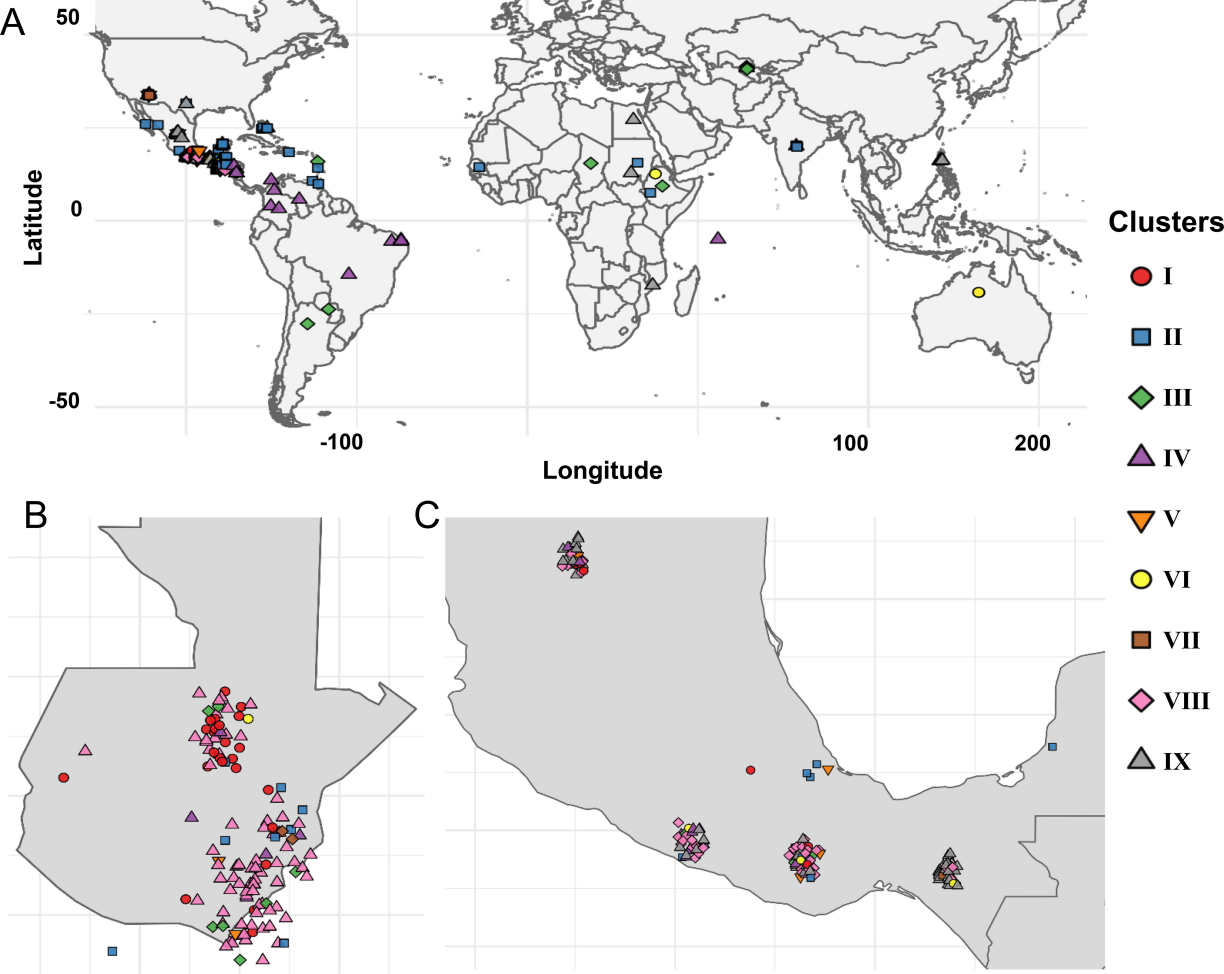
Spatial distribution of cotton landraces across geographic regions. **(A)** Global map showing landrace locations, with color and shape codes representing clusters identified through Neighbor-Joining analysis. Zoomed-in view of landraces originating from Guatemala **(B)** and Mexico **(C)**.

Genotypes in Clusters I and III were primarily found in the highlands of Guatemala and southern Mexico. Cluster VIII accessions mapped exclusively within the Guatemalan highlands, particularly in localities such as Santa Rosa, Huehuetenango, and Alta Verapaz. Cluster V genotypes, composed of cultivated US varieties, were distributed across the US cotton belt. Cluster VI accessions were concentrated in the highlands of Oaxaca, Guerrero, Chiapas, and parts of Guatemala and El Salvador (**Figure 5**). Cluster VII spanned a wide geographic range, including regions in Central America, Africa, Asia, and the Caribbean. Cluster IX showed the broadest geographic spread, with IX-A confined largely to Central America and IX-B extending into North America, Asia, and Caribbean islands.

Analysis of the flowering behavior of landraces in each cluster revealed that photoperiod responses strongly aligned with their genetic groupings (**Supplementary Table S1**). For example, Cluster V, composed primarily of cultivated lines and U.S. landraces, included 31 photoperiod-insensitive genotypes out of 34. Cluster IX, which contains two subclusters, also showed a predominance of insensitive genotypes, with 113 out of 150 accessions requiring multiple growing seasons to flower.

In contrast, Cluster VI which is largely composed of landraces from the Mexican and Central American highlands, had 46 photoperiod-sensitive genotypes out of 48. Cluster VIII showed a similar trend, with 23 out of 29 genotypes from the highlands classified as photoperiod sensitive. Cluster VII included 44 sensitive and 12 insensitive genotypes, while Cluster IV comprised 26 sensitive and 7 insensitive accessions.

Clusters I, II, and III displayed a more balanced mix. Cluster I included five photoperiod-insensitive and four sensitive genotypes. Cluster II contained four photoperiod-insensitive and one sensitive accession. Cluster III comprised three photoperiod-insensitive and two sensitive genotypes.

### STRUCTURE populations and NJ clusters associated with flowering variation in cotton

STRUCTURE analysis of the landrace panel resolved four principal ancestries (Populations I–IV). Integration of these ancestries with NJ clustering and flowering classifications revealed clear associations between genetic ancestry, regional adaptation, and phenological behavior.

Population I comprised 114 genotypes distributed across Central and North America, Asia, Africa, South America, the Caribbean, and Europe (**Figure 3C**). These genotypes drew ancestry from multiple NJ clusters, predominantly IX-A, but also V, IV, I, III, VI, and VII, and included all five modern cultivars, as well as TM-1. Of these, 91 accessions were photoperiod-insensitive and 23 were sensitive (**Figure 3D**).

Population II contained 49 genotypes, primarily originating from the Mesoamerican–Caribbean basin, with additional representatives from Africa, Asia, South America, Australia, and Europe (**Figure 3C**). Nearly all genotypes grouped under Cluster VII. Forty-one accessions were photoperiod-sensitive, and eight were insensitive (**Figure 3D**).

Population III included 107 genotypes, mainly landraces from the Central American and southern Mexican highlands (**Figure 3C**). Most genotypes grouped under Cluster IX-B, with minor contributions from Clusters II and VIII. Flowering behavior was mixed across accession in this population, with 63 photoperiod-sensitive and 44 insensitive genotypes (**Figure 4D**).

Population IV comprised 99 genotypes spanning Central and South America, North America, Asia, Africa, Europe, and Australia (**Figure 3C**). Ancestry was diverse, with major contributions from Clusters IV, VI, and VIII, and additional input from Clusters II, III, and VII. Eighty-two accessions were photoperiod-sensitive, and 17 were insensitive (**Figure 4D**).

Across the full panel, photoperiod-insensitive genotypes showed a strong genetic association with Population I, averaging 44.05% ancestry from this group. In contrast, photoperiod-sensitive genotypes were more closely associated with Populations IV and II, averaging 35.45% and 26.00% ancestry, respectively.

Among individuals with mixed ancestry, photoperiod-insensitive genotypes assigned to Population I tended to have high membership coefficients for that population, indicating a strong genetic identity with Population I. Conversely, sensitive genotypes within Population I showed elevated membership coefficients for Population IV, often exceeding 35%, suggesting admixture with ancestries linked to photoperiod sensitivity.

In Population II, the dominance of photoperiod-sensitive genotypes corresponded to nearly fixed ancestry proportions, reinforcing the strong link between this genetic background and sensitivity to photoperiod (**Figures 3B–D**).

### Genome-wide patterns of Tajima’s D reveal chromosomal variation between cotton landraces and cultivars

**Figure 6:**
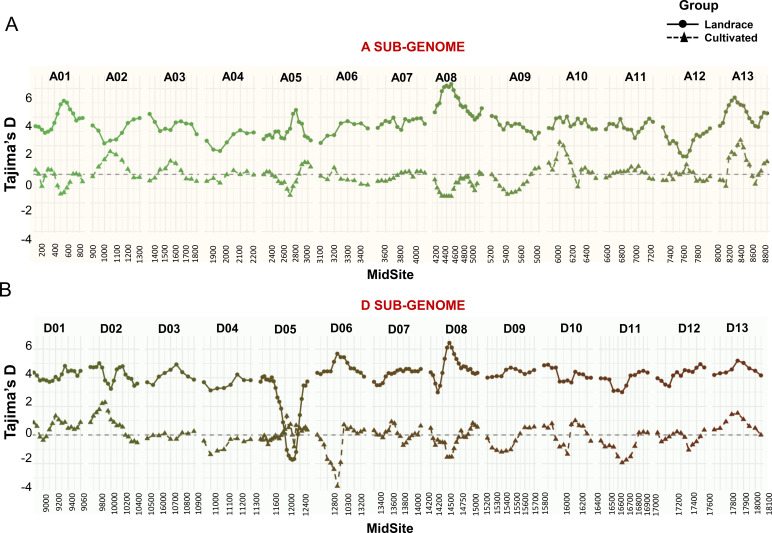
Chromosome-wise estimation of Tajima’s D values for the cotton landrace panel and cultivated germplasm. The values for sub-genomes A **(A)** and D **(B)** were calculated using sliding windows of 50 SNPs with a 200 bp step size. MidSite represents the physical midpoint of each SNP-defined window. A0-A13 and D01-D13 represent thirteen chromosomes in the A and D sub-genomes, respectively.

Tajima’s D values revealed distinct genomic patterns between the landraces and cultivated genotypes. In the landrace panel, Tajima’s D values were predominantly positive, with chromosomes A01, A05, A08, and A13 in the A sub-genome exhibiting sustained peaks ranging from +4.0 to +6.0 (**Figure 6**). Similarly, D sub-genome chromosomes D01, D03, and D08 also showed elevated Tajima’s D values.

In contrast, cultivated accessions displayed Tajima’s D values that were near zero or negative across most of the genome. Notably, negative values were observed on chromosomes A01, A04, and D03, where Tajima’s D values were consistently below zero (**Figure 6**). Analyses were performed using 374 landrace and 6 cultivated genotypes.

Chromosome D05 showed a trough in the central region among landrace genotypes, with Tajima’s D values reaching as low as –3.5. In contrast, cultivated accessions showed near-neutral Tajima’s D values across the same region on D05.

On chromosome A05, cultivars exhibited a strong domestication sweep signal with sharply negative Tajima’s D values. The landrace panel, however, maintained intermediate to high Tajima’s D values on this chromosome. Additionally, chromosome A12 showed reduced Tajima’s D values in landraces relative to other A sub-genome chromosomes.

## Discussion

Intensive selection for yield and fiber-related traits has driven substantial gains in upland cotton productivity over the past century. However, these improvements have come at the cost of a marked reduction in the crop’s basal genetic diversity. Most modern breeding programs, particularly in the United States, rely on a limited set of founder lines and therefore draw from only a fraction of the species’ original allelic richness (Lubbers and Chee, 2009; Tyagi et al., 2014). This narrowing of the genetic base has raised concerns about the long-term resilience and adaptability of cultivated cotton. In light of these concerns, the present study aimed to assess the extent and structure of genetic diversity remaining within *G. hirsutum* landraces and to evaluate the potential of this diversity as a functional reservoir for broadening the genetic foundation of modern cultivars.

In the present study, the depth and quality of the SNP dataset used provided a strong foundation for interpreting population structure, phylogenetic relationships, and potential selective pressures within the landrace panel. High call rates, elevated PIC, and a favorable distribution of MAF indicated a high level of marker informativeness. These characteristics supported robust diversity estimates and enabled fine-scale resolution of genetic relationships across the landrace accessions. The presence of rare alleles further enhanced the analytical power of the dataset, allowing for the detection of low-frequency variants potentially relevant to local adaptation or agronomic performance (Anderson et al., 2010).

The level of marker informativeness observed in the study exceeds those reported in studies focused on elite breeding materials. For instance, a genetic diversity study using the CottonSNP63K array reported average PIC values of 0.17 in a panel of U.S. cultivars (Hinze et al., 2017). Similarly, PIC values around 0.16 were observed in a diversity panel comprising modern upland cotton lines from structured breeding programs (Gowda et al., 2023). In contrast, the PIC value of 0.32 in our study is consistent with those reported for panels enriched with landraces from Central America and genotyped with SSR markers (Shim et al., 2019).

The higher PIC observed in our dataset reflects the inclusion of a diverse set of landraces from across the Americas and other regions, which have retained greater ancestral allelic variation due to limited selection bottlenecks and ongoing adaptation to local environments (Wendel et al., 2010). In contrast, elite breeding programs often prioritize specific alleles associated with yield, fiber quality, or disease resistance, resulting in reduced overall allelic diversity and lower marker informativeness. A higher PIC value indicates that the SNP panel is effective in distinguishing genetic differences among genotypes and in detecting population substructure, both critical features for identifying novel alleles with potential utility in crop improvement. These findings underscore the value of conserving and utilizing landrace materials, not only for their genetic richness but also for their analytical utility in high-resolution genomic studies.

Analysis of genetic distances and population structure revealed that cotton landraces are genetically structured into distinct clusters that often correspond to geographic origin. Notably, landraces from Guatemala, southern Mexico, and Colombia harbored an abundance of rare variants and private alleles. Landraces from these regions also exhibited pronounced genetic divergence from elite cultivars, consistent with previous findings (Wendel et al., 2010). Such divergence underscores the potential of these landraces as reservoirs of unique alleles absent from modern breeding pools.

Variation in within-group genetic distances further highlights the heterogeneity among the inferred populations. For instance, Population I showed moderate internal diversity (mean pairwise distance: 207.96), while Population II exhibited the lowest within-group distance (141.88), indicative of strong genetic cohesion and limited admixture. In contrast, Population IV displayed relatively high internal variation (198.98), suggesting a more complex evolutionary history. These metrics provide additional resolution into the evolutionary trajectories and breeding relevance of each population.

Population II, which includes many Mesoamerican accessions, was the most genetically differentiated from other groups (F_ST_ > 0.65), despite its low internal diversity. This pattern suggests long-term isolation and local adaptation, potentially driven by unique domestication pressures. Conversely, Population IV, comprising accessions from the Caribbean, South America, and Southeast Asia, exhibited greater within-group diversity and lower inter-population differentiation. This is consistent with historical admixture and germplasm exchange (Gowda et al., 2023).

The combined evidence from F_ST_, Nei’s genetic distances, PCA, and NJ clustering supports the conclusion that Mesoamerican and Central American landraces represent deeply divergent lineages. These lineages likely reflect independent domestication events and restricted gene flow, reinforcing their value for cotton improvement. Importantly, the distinct allelic compositions of these landraces highlight their potential as sources of novel genetic variation for traits not captured in elite cultivars.

While STRUCTURE analysis provided a broad overview of population ancestry, the NJ tree offered finer resolution of genetic relationships by incorporating pairwise genetic distances. This approach enabled examination of both broad population assignments and detailed genetic architecture shaped by geography, phenology, and historical gene flow. The distinctness of Cluster IX points to a genetically unique subpopulation, likely maintained through localized cultivation within the Mesoamerican domestication center. In contrast, the heterogeneous composition of Clusters II and VIII suggests historical admixture and gene flow, potentially facilitated by germplasm exchange and breeding activities. The coexistence of region-specific and admixed clusters underscores the complex evolutionary history of upland cotton landraces, shaped by both geographic isolation and human-mediated dispersal.

The NJ tree revealed clusters that were clearly defined by geographic origin (**Figure 4**), a pattern consistent with expectations based on domestication history. Genotypes from the same region often retain shared genetic features due to localized domestication, farmer selection, and adaptation to similar environmental pressures. For example, landraces from the Mexican and Guatemalan highlands clustered tightly within Clusters I, III, VI, and VIII. These groups exhibited limited admixture and strong internal cohesion, suggesting genetic continuity shaped by geographic isolation and traditional cultivation systems. Similarly, Cluster V which included all elite cultivars, TM-1, and U.S. landraces reflected genetic uniformity resulting from intensive breeding programs. These patterns align with previous studies indicating that both regional origin and domestication pathways play key roles in structuring diversity in upland cotton (Wendel et al., 1992; Brubaker and Wendel, 1994; Hinze et al., 2017).

Despite this strong geographic pattern, the NJ tree also captured evidence of historical admixture. Cluster VII, for instance, exhibited high internal diversity and included accessions from diverse regions, including Central America, Africa, Asia, and the Caribbean. This cluster likely reflects complex exchange networks arising from colonial trade and more recent international germplasm movement (Hutchinson et al., 1947; Viot and Wendel, 2023). A more specific example of historical gene flow is observed in Cluster IX-B, where landraces from the Philippines clustered closely with Mexican accessions (**Figure 4**). This unexpected relationship supports historical accounts of the Manila-Acapulco Galleon Trade between 1565 and 1815, which linked Spanish colonies in Mexico and the Philippines and facilitated the exchange of crops, including cotton (Schurz, 1939; Warren, 2012). Such clustering of geographically distant genotypes underscores the significant role of human-mediated dispersal in shaping the genetic landscape of modern cotton.

Notably, the NJ clusters also corresponded with variation in flowering behavior across the landrace accessions. Clusters composed of landraces from the highlands and tropical environments, such as Clusters VI and VIII, were enriched with photoperiod-sensitive accessions. These genotypes are typically late flowering reflecting local adaptation to long-season, rainfed agricultural systems. In contrast, Cluster V and subcluster IX-A were dominated by photoperiod-insensitive accessions, which flower rapidly and predictably under a range of conditions. This trait is a hallmark of modern breeding programs, where early and uniform flowering is critical for commercial cultivation (Lin et al., 2021; Zhou et al., 2022). Intermediate clusters such as Cluster III displayed a mixture of flowering types, suggesting either transitional forms or ongoing introgression between landrace and improved backgrounds.

Beyond its strong association with phylogenetic groupings, flowering behavior in the landraces also showed a clear relationship with genetic ancestry (**Figures 3B-D**). Photoperiod-insensitive accessions were predominantly associated with Population I ancestry, which includes all modern cultivars and closely related landraces. In contrast, photoperiod-sensitive accessions which are characterized by extended flowering periods and reliance on specific day lengths to initiate reproduction shared strong ancestry and grouped in Populations II and IV. These two populations represent traditional landraces that have retained longer vegetative phases and more flexible flowering patterns. This relationship persisted even within admixed populations. For example, several photoperiod-sensitive accessions in Population I carried higher contributions from Population IV ancestry, suggesting that introgressed genomic segments from older landraces influence flowering behavior. Similarly, most individuals in Population II were strongly photoperiod-sensitive and exhibited near-pure ancestry from that group. These findings are consistent with previous reports linking population structure in cotton to adaptive traits such as flowering time (Gowda et al., 2023; Hinze et al., 2015; Hinze et al., 2017).

Flowering patterns did not always align with geographic origin, indicating that genetic background is also a key player in shaping phenology. Comparable associations between flowering time, population structure, and domestication history have been documented in other crops, including maize, barley, and rice (Flint-Garcia et al., 2003; Cockram et al., 2007; Wang et al., 2014). This has practical implications for breeding. Specifically, identifying genomic regions associated with flowering time and photoperiod response can guide the selection of parent lines for specific environments. For instance, cultivars for temperate climates benefit from early, uniform flowering, whereas landraces from tropical regions may provide alleles that confer adaptability to longer or less predictable growing seasons. Collectively, these results highlight the influence of genetic structure and ancestry on flowering behavior and underscore their relevance to targeted cotton improvement.

Beyond population structure and allele frequencies, we examined patterns of nucleotide variation to infer the evolutionary dynamics and selection pressures acting on different cotton gene pools. Genome-wide estimates of Tajima’s D revealed a clear contrast between landraces and elite cultivars, although only six cultivars were analyzed. Landrace populations consistently exhibited positive Tajima’s D values across several chromosomes, suggesting an excess of intermediate-frequency alleles indicative of balancing selection or persistence of long-standing population structure. Such patterns are likely shaped by traditional farming systems, diverse agroecological pressures, and limited artificial selection (Wendel and Cronn, 2003), all of which promote the retention of multiple adaptive alleles and the maintenance of ancestral diversity. In contrast, modern cultivars displayed Tajima’s D values near zero or negative across most of the genome, with notably negative values on chromosomes A01, A04, and D03 (**Figure 6**). These signatures are consistent with directional selection and selective sweeps, likely resulting from intensive breeding aimed at improving yield, uniform flowering, and fiber quality. The reduction in genetic diversity in these regions reflects the effects of modern breeding pipelines, which promote rapid fixation of beneficial alleles but also lead to the loss of rare variants and overall genomic heterogeneity (Wu et al., 2019).

Of particular interest were the contrasting Tajima’s D patterns observed on chromosomes D05 and A05, both enriched for flowering-time genes. Chromosome D05 contains at least 34 flowering-related genes, including *VOZ1* and *PIE1*, both known regulators of flowering time and involved in epistatic interactions shaping phenology (Li et al., 2022; Gowda et al., 2023). In our dataset, landrace accessions exhibited markedly negative Tajima’s D values in this region, consistent with a selective sweep. Such reductions in Tajima’s D are indicative of directional selection, where alleles conferring adaptive values such as those controlling flowering time become rapidly fixed in response to cultivation-specific pressures. For landraces, this likely reflects historical selection for photoperiod responsiveness, enabling synchronization of flowering with regional rainfall patterns and daylength under traditional agroecological regimes. Although not subjected to modern breeding, landraces were still shaped by farmer selection, and these localized sweeps may represent ancient domestication events or adaptation to marginal environments. In contrast, cultivated genotypes exhibited neutral or near-zero Tajima’s D values in the same region, suggesting that D05 has not been a primary target of recent improvement efforts. The presence of negative Tajima’s D values in landraces, particularly in flowering gene regions, underscores their adaptive refinement under specific environmental conditions and highlights their value as a source of functionally selected alleles for improving phenological traits in modern cotton.

On chromosome A05, modern cultivars exhibited sharply negative Tajima’s D values, consistent with a selective sweep likely driven by directional selection during breeding. This region has been a major target for improving flowering time and photoperiod insensitivity, traits essential for adapting cotton to environments with short or fixed growing seasons. Within this region lies *GhCAL*, a key regulator of the plant transition from the vegetative to the reproductive phase. *GhCAL* is known to be upregulated in early flowering cultivars and shares functional similarity with the rice gene *OsbHLH068*, which influences both flowering time and plant height (Zhou et al., 2022; Li et al., 2021). The marked reduction in diversity among cultivars suggests strong artificial selection acting on or near *GhCAL*. In contrast, landrace accessions retained neutral or slightly positive Tajima’s D values across this region, indicating the maintenance of allelic diversity. This preserved variation may confer broader environmental responsiveness, particularly in landraces cultivated under traditional systems with variable photoperiod and rainfall. The divergence in nucleotide diversity between landraces and cultivars at A05 illustrates how modern selection for agronomic uniformity has narrowed genetic variation in key flowering-time loci, while landraces continue to harbor valuable adaptive diversity.

Our findings highlight cotton landraces as a critical source of adaptive genetic variation for modern breeding. They harbor alleles for traits such as drought tolerance, disease resistance, and phenological plasticity, attributes that are becoming increasingly important under climate variability and evolving agricultural systems. Preserved through centuries of farmer selection and adaptation to diverse environments, these landraces retain valuable alleles diminished in modern breeding. Integrating this diversity into elite lines through marker-assisted selection, genomic prediction, and targeted introgression offers a practical pathway to developing cotton varieties with greater resilience and adaptability in the decades ahead.

## Data Availability

The original contributions presented in the study are included in the article/**Supplementary Material**. Further inquiries can be directed to the corresponding author/s.
