# Supplementary material for: Environmental pressures shape regional patterns of genetic diversity and ancestry in cotton landraces
Source: Front Plant Sci. 2025 Nov 21;16:1707011. doi: 10.3389/fpls.2025.1707011 (PMC12678357; doi:10.3389/fpls.2025.1707011)
Supplement: Supplementary file 1 [file Presentation1.pptx]

## Slide 1
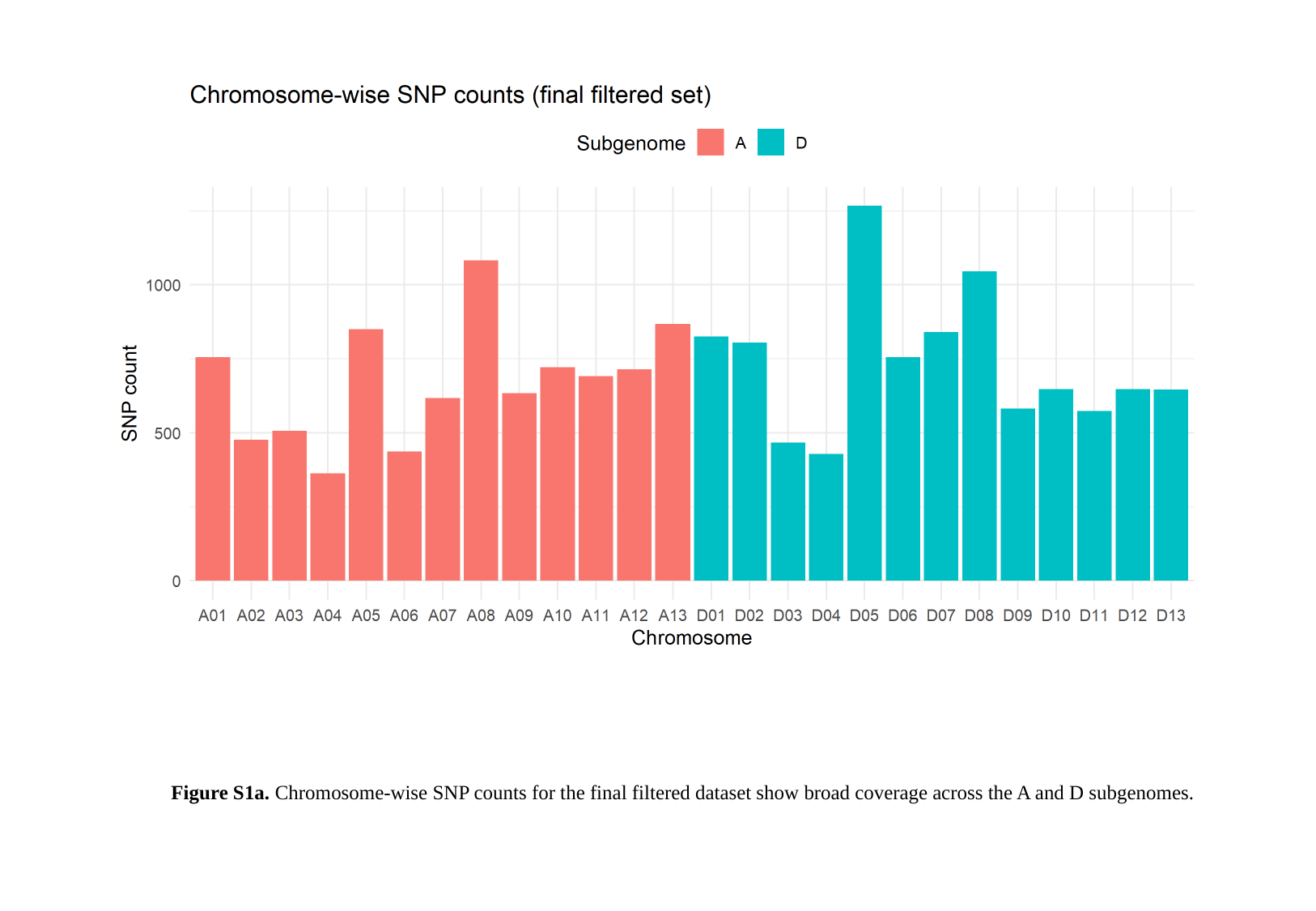

Figure S1a. Chromosome-wise SNP counts for the final filtered dataset show broad coverage across the A and D subgenomes.

## Slide 2
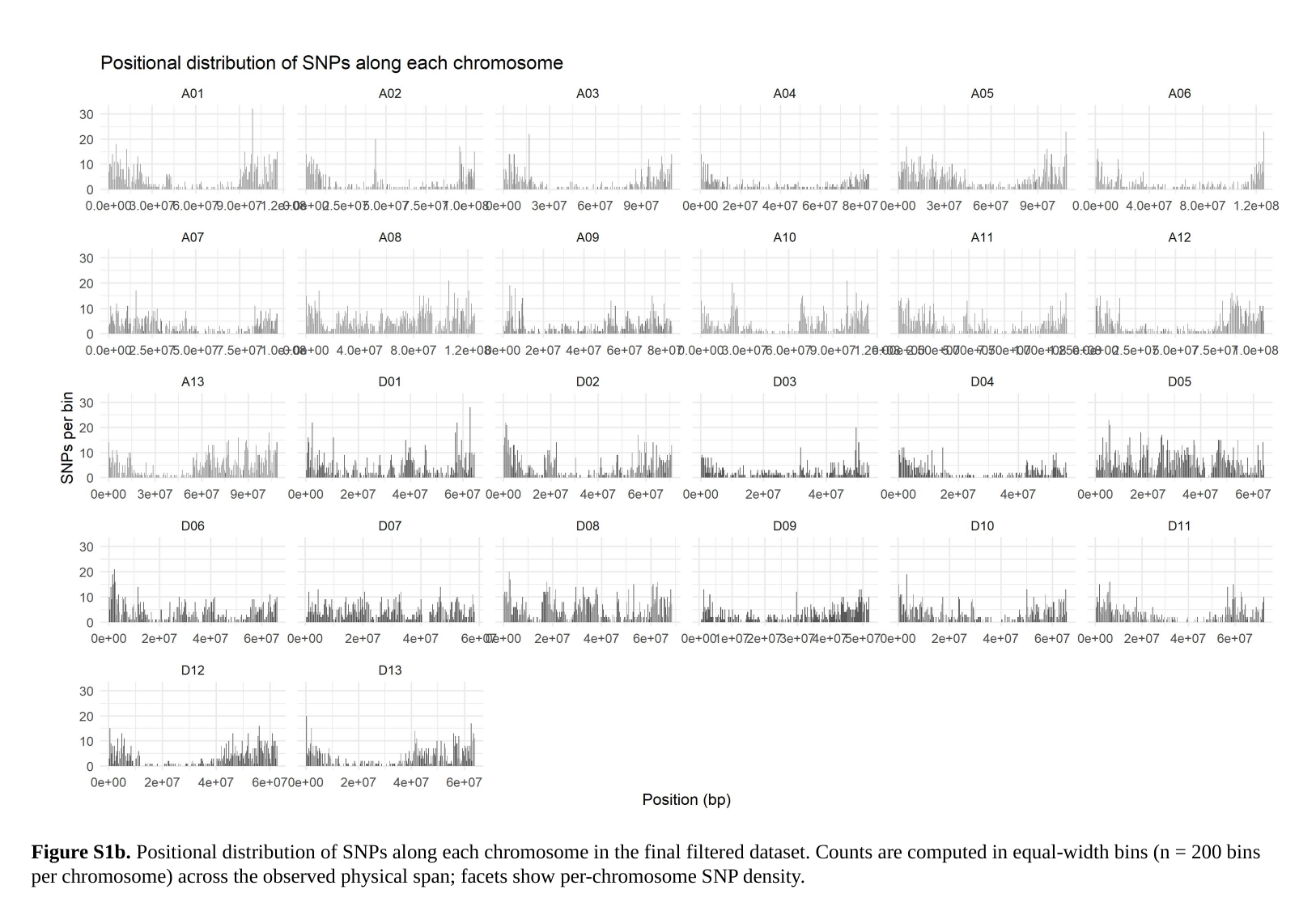

Figure S1b. Positional distribution of SNPs along each chromosome in the final filtered dataset. Counts are computed in equal-width bins (n = 200 bins per chromosome) across the observed physical span; facets show per-chromosome SNP density.
