# Supplementary material for: Environmental pressures shape regional patterns of genetic diversity and ancestry in cotton landraces
Source: Front Plant Sci. 2025 Nov 21;16:1707011. doi: 10.3389/fpls.2025.1707011 (PMC12678357; doi:10.3389/fpls.2025.1707011)
Supplement: Supplementary file 2 [file Presentation2.pptx]

## Slide 1
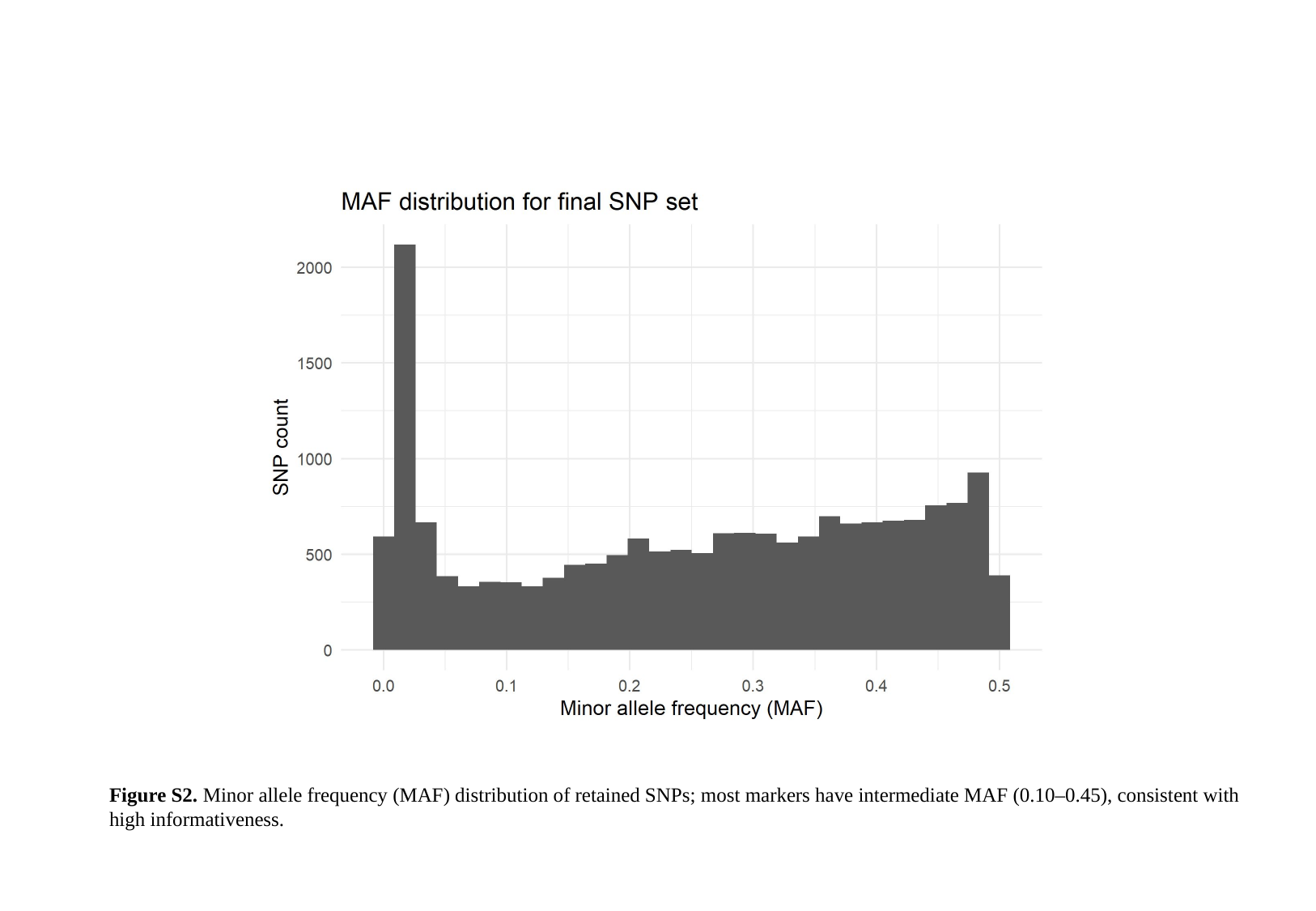

Figure S2. Minor allele frequency (MAF) distribution of retained SNPs; most markers have intermediate MAF (0.10–0.45), consistent with high informativeness.
